# Premenstrual Syndrome, Ultra‐Processed Food Intake, and Food Cravings: A New Perspective

**DOI:** 10.1002/fsn3.70520

**Published:** 2025-06-29

**Authors:** Mahmut Bodur, Nursena Ersoy‐Söke, Emine Karademir, Beyzanur Özkan, Aslı Uçar

**Affiliations:** ^1^ Department of Nutrition and Dietetics, Faculty of Health Sciences Ankara University Ankara Turkey; ^2^ Department of Nutrition and Dietetics, Graduate School of Health Sciences Ankara University Ankara Turkey; ^3^ Department of Nutrition and Dietetics, Bor Faculty of Health Sciences Nigde Omer Halisdemir University Nigde Turkey; ^4^ Department of Medical Biochemistry, Faculty of Health Sciences University of Health Sciences Istanbul Turkey

**Keywords:** food cravings, menstrual health, premenstrual syndrome, ultra‐processed foods

## Abstract

To investigate the relationship between premenstrual syndrome (PMS) prevalence, ultra‐processed food (UPF) consumption, and food cravings in young adult women. A cross‐sectional study was conducted over one menstrual cycle, utilizing the Premenstrual Syndrome Scale to assess PMS symptoms and the Food Craving Questionnaire‐Trait to evaluate food cravings. Dietary data, including UPF consumption, were collected through self‐reported dietary records. The study was carried out among young adult women, focusing on their dietary behaviors and menstrual health. A total of 230 women participated in the study, with a mean age of 20.6 ± 1.8 years. The prevalence of PMS was 61.3% among the participants. Women with PMS reported significantly higher UPF consumption and increased food craving scores compared to those without PMS. UPF energy intake during the menstrual phase was significantly higher in women with PMS (1042.0 ± 30.6 kcal vs. 635.6 ± 41.3 kcal, *p* < 0.001). Multiple linear regression analysis identified food cravings (*B* = 0.468, *p* < 0.001) and UPF consumption (*B* = 0.018, *p* = 0.022) as significant determinants of PMS scores. Excessive consumption of UPFs and increased food cravings are associated with PMS symptoms. These findings highlight the necessity of considering menstrual‐related variations in dietary interventions, particularly with regard to the consumption of UPF and the management of cravings.

## Graphical abstract

This study examines the association between premenstrual syndrome (PMS), ultra‐processed food (UPF) intake, and food cravings in young women. Individuals with PMS reported higher UPF consumption and significantly greater food craving scores across all menstrual phases. The findings highlight the role of dietary patterns in PMS severity and suggest that reducing UPF intake and improving diet quality may support symptom management.

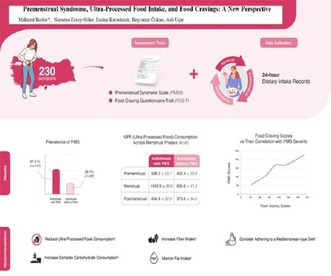

**Abbreviations**
BMI: body mass index
FCQ‐T: Food Craving Questionnaire‐Trait
PMS: premenstrual syndrome
PMSS: Premenstrual Syndrome Scale
SD: standard deviation
SE: standard error
SPSS: Statistical Package for Social Sciences
UPF: ultra‐processed foods

## 1 Introduction

Premenstrual syndrome (PMS) is manifested by a variety of physical, emotional, and psychological symptoms that occur during the luteal phase of the menstrual cycle. Although these symptoms have a significant impact on women's daily personal activities, they resolve spontaneously without treatment a few days after the onset of menstruation (Hofmeister and Bodden 2016; Ryu and Kim 2015). PMS can profoundly affect a woman's quality of life and social functioning. The severity of PMS symptoms often correlates with the duration of the syndrome. Physical manifestations of PMS, including irritability and muscle, joint, back, and abdominal pain, outweigh psychological symptoms (Kahyaoglu Sut and Mestogullari 2016). Although there are no definitive diagnostic criteria for PMS, several diagnostic scales are used to assess the severity and prevalence of symptoms due to their variability among individuals, the influence of psychological factors, subjectivity, and the lack of specific diagnostic tests (Izadi‐Mazidi et al. 2016). PMS affects a significant proportion of women within the reproductive age. While symptoms predominantly manifest among women aged between 25 and 35 years, they can potentially arise across any age group spanning from puberty to menopause (Bahrami et al. 2021; Dilbaz and Aksan 2021). The prevalence of PMS varies between countries, and 75% of women experience PMS symptoms. Apparently, 3%–8% of these have severe symptoms (Upadhyay et al. 2023). An individual's eating habits and nutritional status may be affected by the presence of PMS. Conversely, PMS may also be caused by an excessive intake of carbohydrates, fats, and protein, as well as an inadequate intake of vitamins and minerals, and is closely related to unbalanced ultra‐processed foods (UPF) consumption. However, these effects may be different for each woman as the PMS symptoms (Masruroh and Muniroh 2021; Purnawati et al. 2020).

UPFs are industrially formulated products that go through multiple processing steps and contain numerous ingredients, including additives, preservatives, sweeteners, emulsifiers, colors, flavors, and other chemicals. They are characterized by their high energy content, extended shelf life, increased glycemic load, and variable levels of micronutrients and fiber. These products are palatable and are often marketed in an exceptionally appealing manner (Marron‐Ponce et al. 2019; Srour et al. 2019). The intake of UPF leads to nutritionally unbalanced diets, which are associated with obesity and the development of various chronic diseases that constitute a significant public health problem (Chen et al. 2020).

Food craving is characterized by an intense and specific desire to consume a particular type of food. Such cravings can result in frequent, uncontrolled consumption of the desired foods, often accompanied by feelings of guilt and shame (Chao et al. 2016). Classical and operant conditioning processes, in which environmental and internal factors are associated with food intake, can result in food cravings. These factors trigger food intake, reinforced by rewarding experiences of pleasure or relief from discomfort (Brockmeyer et al. 2015). The presence of PMS is not only one of the factors that can trigger food cravings, but it is also one of the symptoms that are seen in PMS. The multifaceted nature of PMS, including hormonal changes, neurotransmitter fluctuations, psychological factors, and nutritional requirements, is closely related to the increase in food cravings observed during this period (Abdullah et al. 2021; Houghton et al. 2019). Increased food cravings combined with the convenience and palatability of UPFs can lead to increased consumption of these foods during the premenstrual phase. While this may provide temporary relief from PMS symptoms, it may also be a contributor to unhealthier eating patterns (Huwaida et al. 2022). A comprehensive understanding of this relationship is critical to the development of effective PMS symptom management strategies and the promotion of healthier dietary choices. Based on this, the present study aimed to evaluate the relationship between PMS, UPF consumption, and food cravings.

## 2 Material‐Methods

### 2.1 Study Design

This study was conducted with healthy female individuals at the Faculty of Health Sciences, Ankara University, between December 2023 and March 2024. Participants were recruited through brochures, posters, and flyers located in various locations such as the university cafeteria and gym, as previously described (Bodur et al. 2021). Prior to the study, details were explained to the individuals, and those who agreed to participate were scheduled for appointments and referred to the Nutrition and Dietetics Research Unit. Data were collected face‐to‐face by researchers. All procedures followed were in accordance with the ethical standards of the responsible committee on human experimentation (institutional and national) and with the Helsinki Declaration of 1975, and the research protocol was approved by the Ethics Committee of Ankara University (number: 56786525‐050.04.04/100871). Informed consent was obtained from all patients for being included in the study.

During an initial interview with the participants, sociodemographic characteristics, physical activity levels, and anthropometric measurements were collected. In accordance with the recommendations of the World Health Organization, 150 min of physical activity per week is considered “physically active” (WHO 2024a). Anthropometric data were collected in accordance with standardized procedures to ensure accuracy and reproducibility. Height was measured to the nearest 0.1 cm using a wall‐mounted stadiometer. Participants' body weight was measured using a calibrated digital scale while they were barefoot and wearing light clothing. The values were recorded to the nearest 0.1 kg. BMI was computed as weight (kg) divided by height squared (m^2^). This measure was applied to categorize individuals according to the World Health Organization (WHO) classifications for underweight, normal weight, overweight, and obesity (WHO 2024b). The BMI cut‐off points used in this study were as follows: underweight < 18.5 kg/m^2^, normal weight = 18.5–24.9 kg/m^2^, overweight = 25.0–29.9 kg/m^2^, and obese ≥ 30.0 kg/m^2^. In order to classify menstrual cycle phases, participants were asked to report the first day of their last menstrual period (LMP). A fixed day‐count method was used to define three distinct phases: the premenstrual phase as days −7 to −1 before menstruation onset, the menstrual phase as days 1 to 4 (with day 1 being the first day of bleeding), and the postmenstrual phase as days 5 to 11. This standardized approach was applied consistently to all participants with a view to reducing misclassification bias and enhancing the consistency of data collected across phases. During the premenstrual period, the Premenstrual Syndrome Scale and a Food Craving Questionnaire‐Trait were administered, and a 24‐h dietary recall was recorded to determine food consumption, and the consumption of UPF. A 24‐h food recall was used to assess food consumption and UPF intake during both the premenstrual, menstrual, and postmenstrual phases.

### 2.2 Sample Size and Inclusion Criterias

The sample size required for the study was calculated using Gpower (Faul et al. 2009) (Figure S1). This calculation considered the interactions within and between groups in repeated measures based on the PMS status. Accordingly, with an effect size of 0.10, a Type I error of 0.05, a power of 0.80, two groups, three repeated measurements, and a correlation of 0.3 between measurements, the required sample size was determined to be 228. To account for the potential dropout of participants during the study, it was targeted to include 256 individuals in the research.

This study has specific inclusion criteria: participants must have a regular menstrual cycle, defined as cycles occurring every 22–35 days and lasting 3–8 days; they must not have any diagnosed medical conditions; they must not be taking any medications, vitamins, or drugs; and they must not be on any special diet due to chronic medical conditions. Initially, 256 individuals participated in the study. However, 15 of them discontinued their participation due to non‐compliance with follow‐up procedures, and 11 were excluded from the final dataset due to missing data.

### 2.3 Assessment of Premenstrual Syndrome

The study used the Premenstrual Syndrome Scale (PMSS) to assess the presence of premenstrual syndrome in participants. The PMS scale, which was developed by Gençdoğan in 2006, is based on the criteria of the DSM‐III and DSM‐IV‐R and aims to measure the intensity of premenstrual symptoms. The Turkish validation study reported a Cronbach's alpha coefficient of 0.96 for the total scale, indicating excellent internal consistency. Subscale alpha values ranged from 0.76 to 0.94. The factor structure of the Turkish version was consistent with the original instrument, and confirmatory factor analysis supported its construct validity. These findings provide support for the use of the PMSS in Turkish populations. The PMS consists of 44 items, which individuals rate according to the symptoms they experience in the week before menstruating. The PMS scale comprises nine subcategories: depressive mood, anxiety, fatigue, irritability, depressive thoughts, pain, changes in appetite, sleep variations, and bloating. Although the DSM‐5 no longer classifies PMS as a diagnosis (only PMDD is recognized), the PMSS remains a widely used tool in Turkish research contexts for the assessment of a wider spectrum of premenstrual symptoms. Participants rate each subcategory on a five‐point Likert scale. Participants were dichotomized into PMS and non‐PMS groups based on the validated threshold of the PMSS, where a score exceeding 50% of the maximum total score was used to define the presence of PMS. Higher scores on the scale indicate more severe premenstrual symptoms (Gencdoğan 2006).

### 2.4 Food Craving Questionnaire‐Trait

The Food Craving Scales (FCQ‐T) were developed by Cepeda‐Benito et al. (2000) as self‐report instruments to measure food cravings. In 2019, Akkurt and colleagues conducted a study to validate and assess the reliability of the Turkish version of the FCQ‐T scale (Akkurt et al. 2019). The scale consists of 39 items, with responses measured on a six‐point Likert scale ranging from 1 (never) to 6 (always). To calculate the scores for each sub‐dimension and for the total scale, the sum of the item scores is divided by the number of items. To determine the intensity of food cravings within specific sub‐dimensions, particularly for intense food cravings, the scores of the relevant items are averaged. A higher total score on this scale is an indication of a greater tendency toward excessive food craving. Although the FCQ‐T is a trait‐based instrument, it was administered during the premenstrual phase to reflect the typical timing of increased food cravings. This approach allowed for the examination of the association between trait‐level cravings and the severity of premenstrual symptoms.

### 2.5 Assessment of Dietary Intake and Ultra‐Processed Food Consumption

In order to determine individuals' daily food intake, a 24‐h food intake record was taken face‐to‐face by trained dietitians during each third of each menstrual phase (premenstrual phases, menstrual phases, postmenstrual phases). A photographed food catalog was used to ascertain the type and amount of food/liquids consumed (Rakıcıoğlu et al. 2012). The daily energy and macro and micronutrients of the individuals were calculated using the Nutritional Information System 8.1 software (Pasifik Elektrik Elektronik Ltd. Şti 2021). A single 24‐h dietary recall was collected for each menstrual phase to evaluate phase‐specific dietary intake. This method was preferred over food frequency questionnaires (FFQs) or repeated recalls in order to reduce recall bias and to better capture short‐term dietary variations. While this approach may not fully represent habitual intake, it provided detailed and time‐specific data. All interviews were conducted face‐to‐face by trained dietitians, using a standardized food photography catalog to enhance portion size estimation and data accuracy.

UPFs are assessed according to the NOVA classification. UPFs are classified according to the foods consumed in eight different categories: ultra‐processed breads and breakfast foods, sauces, spreads and condiments, packaged sweet snacks and desserts, packaged savory snacks, sugar and artificially sweetened beverages, meat/poultry/seafood‐based ready meals, ready‐to‐eat/heat mixed meals and dairy‐based desserts (Fang et al. 2024; Khandpur et al. 2021). Foods were classified as ultra‐processed (NOVA Group 4) if they were industrial formulations typically containing five or more ingredients, including substances not commonly used in culinary preparations (e.g., hydrogenated oils, flavor enhancers, emulsifiers, preservatives). Classification was based on the detailed description of each item in the 24‐h recall, including brand names when available. Trained dietitians cross‐referenced all entries with the NOVA criteria to ensure consistent categorization. Common examples included packaged biscuits, soft drinks, instant noodles, flavored yogurts, processed meats, and ready‐to‐eat frozen meals. The consumption of UPFs by individuals has been identified based on their dietary intake records taken over three different times, namely premenstrual, menstrual, and postmenstrual phases. The study quantified the ratio of energy and macronutrients obtained from UPFs to the overall daily dietary intake.

### 2.6 Statistical Analysis

**FIGURE 1:**
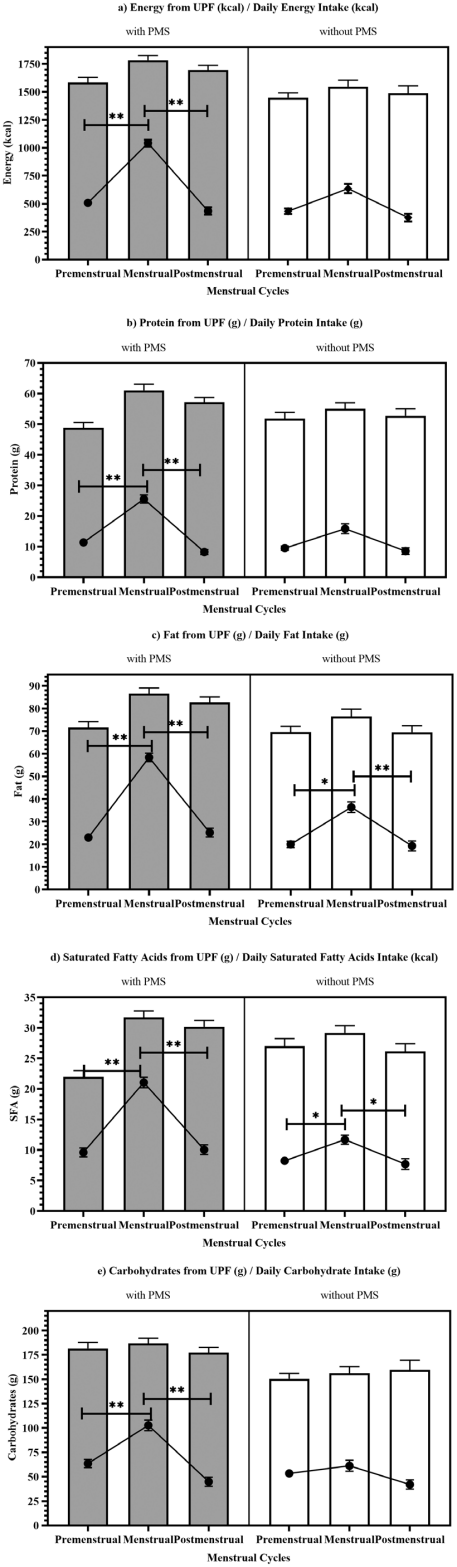
The comparison of macronutrient intake from UPF by PMS status. (a) Energy from UPF (kcal)/daily energy intake (kcal); (b) Protein from UPF (g)/daily protein intake (g); (c) Fat from UPF (g)/daily fat intake (g); (d) Saturated fatty acids from UPF (g)/daily saturated fatty acids intake (g); (e) Carbohydrate from UPF (g)/daily carbohydrate intake (g).

The statistical analysis of the data obtained in the study was performed using the SPSS (Statistical Package for Social Sciences) program. Group differences were evaluated using statistical methods (e.g., *t*‐tests, regression) that do not require equal sample sizes. In addition to group‐based analyses, continuous PMSS scores were used in multiple regression models to preserve statistical power and account for the full range of symptom severity. Descriptive statistical analyses were presented as mean ± standard deviation ($\bar{x}$ ± SD) when numerical data followed a normal distribution. Non‐numerical data were expressed using counts (*n*) and percentages (%). Histograms and statistical tests were used to assess the normality of the data. Table 1 shows the demographic features of participants and comparison of UPF consumption between individuals with and without PMS during different menstrual phases. Figure 1 illustrates the daily dietary proportions of energy and macronutrients derived from UPF consumption during menstrual periods, categorized by individuals' PMS statuses. Table 2 presents a multiple linear regression model showing the relationship between PMS scores, food craving, UPF‐derived energy in menstrual phases, BMI, and age. The model includes adjusted energy, carbohydrate (CHO), and SFA quantities adjusted to 1000 kcal. Statistical significance was determined at *p* < 0.05. BMI was included as a covariate in the regression models (Models 2 and 3). Additionally, interaction terms (BMI × FCQ‐T scores and BMI × UPF energy intake across menstrual phases) were tested, but none of them reached statistical significance (*p* > 0.05), and thus were not retained in the final model.

**TABLE 1:** Comparison of characteristics between individuals with and without PMS.

	PMS syndrome		*u*/*t p*
	With PMS (*n* = 141)	Without PMS (*n* = 89)	
Age (years), *x* ± SD (min–max)^a^	20.6 ± 1.8 (18.0–28.0)	21.4 ± 2.6 (18.0–27.0)	−2.587 0.010*
Smoking status, *n* (%)^b^	6 (4.3)	4 (4.5)	0.007 0.931
BMI (kg/m^2^), $\bar{x}$ ± SD (min–max)^a^	21.5 ± 3.1 (16.4–31.4)	21.5 ± 3.0 (16.2–30.9)	0.062 0.951
BMI categories, *n* (%)^b^			
Underweight	27 (19.1)	15 (16.9)	0.218 0.897
Normal	98 (69.5)	63 (70.8)	
Overweight or obese	16 (11.3)	11 (12.4)	
Physically active, *n* (%)^b^, ^c^	21 (14.9)	20 (22.5)	2.139 0.144
FCQ (points), $\bar{x}$ ± SD (min–max)^a^	145.0 ± 31.8 (94.0–202.0)	75.1 ± 20.1 (33.0–111.0)	23.772 < 0.001**
PMS (points), $\bar{x}$ ± SD (min–max)^a^	136.8 ± 18.5 (110.0–182.0)	81.2 ± 15.2 (44.0–109.0)	18.476 < 0.001**

^a^ Independent sample *t* test were used.

^b^ Chi‐square test was used.

^c^ At least 150 min/week Defined as engaging in ≥ 150 min of physical activity per week, in accordance with WHO guidelines, BMI categories were based on WHO standards: underweight < 18.5 kg/m^2^, normal weight = 18.5–24.9 kg/m^2^, overweight = 25.0–29.9 kg/m^2^, obese ≥ 30.0 kg/m^2^.

* denotes statistical significance at *p* < 0.05.

** denotes *p* < 0.001.

**TABLE 2:** Regression modeling of the food craving and ultraprocessed food consumption.

PMS scores					
Dependent outcome PMS scores	Unstandardized *B*	SE	(% 95 CI lower‐upper)	*p*	*R*/*R* ^2^
Model 1					
Food cravings	0.497	0.036	0.427, 0.567	< 0.001**	0.679/0.461
Model 2					
Age	−0.966	0.724	−2.394, 0.461	0.183	0.685/0.470
BMI	0.754	0.520	−0.270, 1.778	0.148	
Food cravings	0.491	0.036	0.420, 0.562	< 0.001**	
Model 3					
Food cravings	0.468	0.037	0.395, 0.541	< 0.001**	0.694/0.482
Age	−0.767	0.726	−2.197, 0.663	0.292	
BMI	0.810	0.520	−0.214, 1.835	0.120	
Energy intake from UPF in premenstrual term	1.487 × 10^−4^	0.006	−0.012, 0.013	0.981	
Energy intake from UPF in menstrual term	0.018	0.008	0.003, 0.034	0.022*	
Energy intake from UPF in postmenstrual term	6.837 × 10^−4^	0.007	−0.014, 0.015	0.925	

*Note:* Model 1. Crude model (PMS scores = Food Cravings). Model 2. Adjusted for model 1 + Age + BMI. Model 3. Adjusted for model 2 + Energy intake from UPF in premenstrual phase + Energy intake from UPF in menstrual term + Energy intake from UPF in postmenstrual term.

* denotes statistical significance at *p* < 0.05.

** denotes *p* < 0.001.

## 3 Results

Table 1 shows the comparison of the characteristics between individuals with and without PMS. In the study, it was determined that 61.3% (*n* = 141) of the 230 individuals who participated had PMS. The mean age of individuals with PMS was 20.6 years (SD ±1.8, range 18.0–28.0), while the mean age of those without PMS was 21.4 years (SD ±2.6, range 18.0–27.0) (*p* < 0.001). Smoking status was similar in both groups, with 4.3% in the PMS group and 4.5% in the individuals without PMS group. The mean BMI was almost identical in both groups, with values of 21.5 kg/m^2^ (SD ±3.1, range 16.4–31.4) for those with PMS and 21.49 kg/m^2^ (SD ±3.0, range 16.2–30.9) for those without. The distribution of BMI categories (underweight, normal, overweight or obese) was also similar. Physical activity was reported by 14.9% of the PMS group and 22.5% of the individuals without PMS group. The Food Craving Questionnaire (FCQ) scores differed significantly between the groups. Those with PMS had an average score of 145.0 points (SD ±31.8, range 94.0–202.0), while those without PMS had an average score of 75.1 points (SD ±20.1, range 33.0–111.0). Similarly, PMS scores were higher in the PMS group, averaging 136.8 points (SD ±18.5, range 110.0–182.0) compared to 81.2 points (SD ±15.2, range 44.0–109.0) in the individuals without PMS group.

Figure 1 and Table S1 show the daily intake of energy and macronutrients (CHO, Protein, Fat, SFA) from the diet of individuals across menstrual phases, as well as the energy and macronutrients obtained from the consumption of UPF.

In the study, energy intake was consistently higher in PMS across all phases, peaking during the menstrual phase (1784.0 ± 41.0 kcal) compared to those without PMS (1546.8 ± 58.1 kcal). Individuals with PMS consumed more energy from UPF during the menstrual phase (1042.0 ± 30.6 kcal, *p* < 0.001) compared to the premenstrual (508.2 ± 23.7 kcal, *p* = 0.025) and postmenstrual phases (434.9 ± 32.5 kcal, *p* < 0.001). Individuals without PMS showed a higher energy intake from UPF during the menstrual phase (635.6 ± 41.3 kcal, *p* < 0.001) compared to the premenstrual (432.4 ± 23.9 kcal, *p* = 0.227) and postmenstrual phases (375.6 ± 34.0 kcal, *p* < 0.001). The highest carbohydrate intake was observed during the menstrual phase for both groups. Individuals with PMS consumed 186.5 ± 5.3 g (*p* < 0.001), while those without PMS consumed 156.3 ± 6.8 g (*p* = 0.086). Furthermore, carbohydrate intake from UPF was also highest during the menstrual phase. Individuals with PMS consumed 102.7 ± 5.4 g (*p* < 0.001) of UPF, while those without PMS consumed 61.2 ± 5.7 g (*p* < 0.001) of UPF. Similarly, fat intake exhibited a peak during the menstrual phase. İndividuals with PMS consumed 86.5 ± 2.6 g (*p* = 0.001), while those without PMS consumed 76.4 ± 3.3 g (*p* = 0.110). The highest levels of fat intake from UPF were observed during the menstrual phase for both groups. Individuals with PMS consumed 58.4 ± 1.9 g of UPF (*p* < 0.001), while those without PMS consumed 36.4 ± 2.4 g (*p* = 0.046). Furthermore, the highest intake of saturated fats was observed during the menstrual phase. Individuals with PMS showed an average intake of 31.7 ± 1.0 g (*p* = 0.015), while those without PMS had an average intake of 29.2 ± 2.1 g (*p* < 0.001). The intake of saturated fats from UPF showed a peak during the menstrual phase for both groups. Individuals with PMS demonstrated the highest intake at 21.1 ± 0.9 g (*p* < 0.001), while those without PMS exhibited the lowest intake at 7.7 ± 0.8 g (*p* = 0.046). The highest protein intake was recorded during the menstrual phase. Individuals with PMS consumed more protein (61.0 ± 2.0 g) than those without PMS (55.0 ± 2.0 g). This difference was statistically significant (*p* = 0.029). During the menstrual phase, women with PMS consumed significantly more protein from UPF (25.6 ± 1.4 g, *p* < 0.001) compared to those without PMS (15.9 ± 1.6 g, *p* < 0.001).

Table 2 shows regression models that assess the relationship between PMS scores and food cravings. In the unadjusted model (Model 1), food cravings emerged as a significant predictor of PMS scores (*B* = 0.497, SE = 0.036, 95% CI: 0.427, 0.567, *p* < 0.001). Upon adjusting for age and BMI, food cravings continued to significantly predict PMS scores (*B* = 0.491, SE = 0.036, 95% CI: 0.420, 0.562, *p* < 0.001), indicating that the effect of food cravings on PMS scores is robust across different ages and BMIs. Age and BMI were not significant predictors of PMS scores. In model 3, adding energy intake from UPF during different menstrual phases, food cravings remained a significant predictor of PMS scores (*B* = 0.468, SE = 0.037, 95% CI: 0.395, 0.541, *p* < 0.001). Notably, energy intake from UPF during the menstrual term significantly predicted PMS scores (*B* = 0.018, SE = 0.008, 95% CI: 0.003, 0.034, *p* = 0.022), suggesting a specific temporal relationship between UPF consumption and PMS severity. Neither energy intake in the premenstrual (*B* = 0.0001487, SE = 0.006, 95% CI: −0.012, 0.013, *p* = 0.981) nor postmenstrual terms (*B* = 0.0006837, SE = 0.007, 95% CI: −0.014, 0.015, *p* = 0.925) was associated with PMS scores.

## 4 Discussion

This study aimed to evaluate the UPF consumption according to the presence of PMS and menstrual cycle phases in female university students. Moreover, it aimed to explain the relationship between PMS severity and food craving. According to the current literature, no previous study has investigated UPF consumption in relation to PMS and menstrual cycle phases, along with the association between PMS severity and food cravings. The present study results showed that while the energy, macronutrient, and saturated fat intake from UPF differed among the menstrual cycle phases of individuals with PMS, a significant difference was found between fat intake from UPF and saturated fat intake across menstrual cycle phases in individuals without PMS. When BMI and age covariances are taken into account, UPF intake and food craving account for 48.2% of PMS scores.

The individuals with PMS syndrome were of a younger age and showed higher FCQ scores. Although BMI was not a significant predictor in the regression models, it was included as a covariate to control for potential confounding effects. Higher BMI may influence hormonal regulation, systemic inflammation, and reward‐related eating behaviors, all of which may influence PMS symptoms. Additionally, increased adiposity may alter metabolic responses to UPF consumption, such as impaired insulin sensitivity or shifts in nutrient absorption. Although the interaction terms between BMI and craving or UPF intake were tested and found to be non‐significant, these pathways should be investigated further in future research. Furthermore, individuals with PMS consumed a greater amount of UPFs throughout the premenstrual and menstrual phases compared to those without PMS. Consuming more UPF during the premenstrual and menstrual phases may have been preferred to reduce PMS symptoms. However, the consumption of UPF during each menstrual cycle has not previously been investigated. This emphasizes the necessity for more research to explore this association.

Consuming UPF is related to numerous negative health consequences. Consuming UPF is associated with a higher intake of added sugars, salt, and trans fats and an increased energy intake, resulting in a poor nutritional quality (Elizabeth et al. 2020). Moreover, research has established a correlation between elevated consumption of UPF and inadequate nutritional quality among women in their reproductive years (Habibi et al. 2022). Assessing nutrient consumption during the menstrual cycle and PMS becomes more crucial due to the fluctuations in nutrient intake that occur during this period (Cross et al. 2001). Individuals with PMS have a tendency to increase their intake of energy, carbohydrates, proteins, lipids, and saturated fats. Additionally, they consume higher quantities of total sugars, sodium, and trans fats. Individuals with PMS exhibited excessive consumption of energy, carbohydrates, and saturated fats in this study. Researchers have discovered that persons suffering from PMS consume greater amounts of energy, carbohydrates, proteins, lipids, and saturated fats (Houghton et al. 2017; Taheri et al. 2023). The association between increased UPF consumption and increased intakes of energy, added sugars, fat, and saturated fat emphasizes the importance of evaluating UPF consumption in individuals with PMS (Martini et al. 2021).

Moreover, a Western‐style diet that contains excessive amounts of sodium and fat was found to be related to PMS (MoradiFili et al. 2020), whereas a Mediterranean‐style diet was associated with reduced PMS symptoms (Kwon et al. 2022). The Mediterranean diet is linked to the consumption of beneficial fats, complex carbohydrates, and reduced sodium intake (Willett 2006). There is a correlation between the prevalence of PMS and the use of bread/snack dietary pattern, sugar, cola beverages, chocolate, and biscuits (Kwon et al. 2022; Yilmaz et al. 2021; Ünal and Uçar 2024). Furthermore, research has demonstrated that substituting simple carbohydrates with complex carbohydrates can have an impact on the symptoms of PMS in individuals who experience PMS (Esmaeilpour et al. 2019). Individuals with PMS were found to consume a significantly higher amount of carbohydrates during both the premenstrual and menstrual phases. Additionally, they had a high intake of UPF specifically during the menstrual phase. These findings suggest that the type of carbohydrates consumed from UPF sources may have an impact on PMS symptoms. Another research investigation discovered a direct correlation between PMS and protein consumption (Gorczyca et al. 2016). Nevertheless, research indicates that the consumption of protein does not have any impact on PMS (Houghton et al. 2019). Similarly, this study did not reveal any association between premenstrual syndrome and dietary protein consumption.

This study assessed the consumption of nutrients and UPF not only during the presence of PMS but also during menstrual phases. Furthermore, the assessment of UPF consumption during the premenstrual, menstrual, and postmenstrual phases represents a novel viewpoint. Gallon et al. examined the overall energy consumption and carbohydrate consumption during the follicular and luteal phases, as well as the differences between these phases based on the situation of PMS. Individuals with PMS exhibited increased levels of energy and consumed more carbohydrates; however, individuals without PMS did not demonstrate any difference when comparing the luteal phase with the follicular phase. In addition, the comparison of the luteal and follicular phases in individuals with and without PMS in this study revealed that individuals with PMS had elevated levels of energy and carbohydrate consumption throughout the luteal phase (Gallon et al. 2022). In individuals with PMS, the premenstrual period showed higher intakes of CHO, complex CHO, protein, free sugars, fat, and energy compared to the postmenstrual period. Only energy intake increased during the premenstrual period in individuals without PMS. Cakes and sweets, foods high in sugar, drinks, and salty snacks were found to be higher in the premenstrual period (Cross et al. 2001). Individuals with PMS experienced elevated levels of energy, carbohydrates, proteins, fats, and saturated fats during their menstrual phase compared to other phases. However, in individuals without PMS, only fat and saturated fat intake from UPF in the menstrual phase is higher than in the premenstrual and postmenstrual phases. Compared to those without PMS, those with PMS consumed more carbohydrates and energy from UPF during the premenstrual period. In this study, the increase in UPF intake supports the increase in nutrient intake during the menstrual phase.

The regression analysis revealed a significant association between the severity of PMS and both food cravings and energy intake from consuming UPF during the menstrual phase. Individuals with PMS exhibit elevated desires for foods that are rich in sugar or fat (Yen et al. 2010). This could be attributed to decreased serotonin activity in the premenstrual phase. Furthermore, hormonal fluctuations can cause food cravings in females as a result of increased energy expenditure or inadequate nutrition, particularly in the presence of negative emotions. Food cravings may occur, particularly during PMS and the luteal phase. Food cravings, particularly in PMS, can be noted during the luteal phase, and diet consumption has been linked to an increase in appetite (Dye and Blundell 1997). However, since food craving was assessed once in this study, it could not be assessed between phases. In addition, in this study, it was determined that the highest energy intake of individuals was in the menstrual phase.

The consumption of UPF may also play a significant role in food cravings and the severity of PMS. An Iranian study has found that consuming food high in calories, fat, sugar, and salt is related to a higher likelihood of experiencing PMS (Hashim et al. 2019). Similarly, a study conducted in India on girls aged 11 to 19 found that girls who frequently consumed an unhealthy diet had a higher occurrence of PMS symptoms (Vani et al. 2013). Moreover, studies have shown a direct relation between a diet that is characteristic of Western culture, which is rich in sodium and fat, and the severity of PMS symptoms in individuals aged 20 to 45 (Farasati et al. 2015). Although the literature does not specifically investigate the severity of PMS using the phrase “ultra‐processed foods” (UPF), it does recognize junk food and high‐fat sugary meals as examples of UPF (Elizabeth et al. 2020; Christ et al. 2019). Additionally, the western‐style diet is known to include a substantial quantity of UPF. Therefore, the results of this study can be interpreted in light of the previously mentioned study results on this subject. Reducing the intake of high‐calorie, fatty, sugary, and salty meals may potentially lead to a decrease in PMS symptoms by converting estrogen into its inactive state (Hashim et al. 2019). In addition, adhering to low‐fat and high‐fiber diets can potentially improve symptoms of PMS by reducing the amounts of estrogen sulfate in the bloodstream. Insufficient intake of magnesium from consuming increased amounts of sugary or fatty foods may worsen the severity of premenstrual syndrome (Farasati et al. 2015).

### 4.1 Limitations

It is important to note that this study also has potential limitations. Firstly, causality was not determined due to the cross‐sectional design of the study. Secondly, UPF consumption was calculated using the 24‐h dietary recall method. Therefore, there may be deficiencies in remembering. Moreover, since only one recall was conducted per phase, day‐to‐day variability and the episodic nature of UPF consumption may not be fully captured. This may lead to potential underestimation or misreporting of food intake. In future investigations, UPF consumption can be assessed using a valid and reliable tool. Additionally, several potential confounding factors, such as stress levels, sleep quality, socioeconomic status, and detailed physical activity intensity, were not measured or included in the analyses. These variables could have influenced PMS severity and dietary behaviors, and their absence limits the comprehensiveness of the model. Future studies should aim to incorporate these factors within longitudinal or repeated‐measures designs to better elucidate causal pathways. Food cravings were assessed only once using the FCQ‐T, which evaluates general craving tendencies rather than phase‐specific fluctuations. This is a limitation in assessing dynamic changes in cravings across the menstrual cycle. Finally, the study included the assessment of premenstrual, menstrual, and postmenstrual phases during a single menstrual cycle. It should be recognized that different results may be obtained during other cycles. Although we used a validated cut‐off to classify participants into PMS and non‐PMS groups, we acknowledge that dichotomizing a continuous score may obscure more subtle variations in symptom severity. Future research may benefit from analyzing PMS as a continuous outcome to retain more statistical power and nuance. The PMSS used in this study is based on earlier DSM‐III/DSM‐IV‐R criteria. While it captures a wide range of PMS‐related symptoms, it is not aligned with the current DSM‐5 diagnostic framework, which only recognizes PMDD. This should be considered when interpreting the clinical implications of the results.

## 5 Conclusion

In conclusion, food cravings and UPF may also play a significant role in PMS severity. Targeted nutritional counseling focused on reducing UPF consumption may serve as a non‐pharmacological approach for alleviating premenstrual syndrome symptoms. Emphasizing dietary awareness and behavior regulation during the premenstrual phase could support symptom management. Further longitudinal studies are recommended to evaluate the causal relationships and effectiveness of such dietary interventions.

## Author Contributions

**Mahmut Bodur:** conceptualization (lead), data curation (lead), methodology (lead), writing – original draft (equal), writing – review and editing (equal). **Nursena Ersoy‐Söke:** data curation (equal), investigation (equal), writing – original draft (equal), writing – review and editing (equal). **Emine Karademir:** data curation (equal), investigation (equal), writing – original draft (equal), writing – review and editing (equal). **Beyzanur Özkan:** data curation (equal), investigation (equal), writing – original draft (equal), writing – review and editing (equal). **Aslı Uçar:** conceptualization (equal), project administration (equal), supervision (equal), writing – review and editing (equal).

## Disclosure

The authors have nothing to report.

## Conflicts of Interest

The authors declare no conflicts of interest.

## Supporting information

Supplementary files.

## Data Availability

The data that support the findings of this study are available from the corresponding author upon reasonable request.
