# Supplementary material for: Premenstrual Syndrome, Ultra‐Processed Food Intake, and Food Cravings: A New Perspective
Source: Food Sci Nutr. 2025 Jun 29;13(7):e70520. doi: 10.1002/fsn3.70520 (PMC12206854; doi:10.1002/fsn3.70520)
Supplement: Supplementary file 1 — Supplementary files. [file FSN3-13-e70520-s001.docx]

**Figure S1.** Power analysis of the study

**F tests -** ANOVA: Repeated measures, within-between interaction

**Analysis:** A priori: Compute required sample size

**Input:** Effect size f = 0.10

α err prob = 0.05

Power (1-β err prob) = 0.80

Number of groups = 2

Number of measurements = 3

Corr among rep measures = 0.3

Nonsphericity correction ε = 1

**Output:** Noncentrality parameter λ = 9.7714286

Critical F = 3.0156752

Numerator df = 2.0000000

Denominator df = 452

Total sample size = 228

**Table S1.** Ultra-processed food consumption through the menstrual cycles

|  | **PMS** | | | | | |  |  |  |  |
| --- | --- | --- | --- | --- | --- | --- | --- | --- | --- | --- |
|  | **With PMS (n=141)** | | | **Without PMS (n=89)** | | |  |  |  |  |
|  | **Premenstrual** | **Menstrual** | **Postmenstrual** | **Premenstrual** | **Menstrual** | **Postmenstrual** |  |  |  |  |
| **UPF consumption**^†^ |  |  |  |  |  |  | **p^a^** | **p^b^** | **p^c^** | **p^d^** |
| Energy (kcal) | 508.2±23.7 | 1042.0±30.6 | 434.9±32.5 | 432.4±23.9 | 635.6±41.3 | 375.6±34.0 | 0.025 | <0.001 | 0.227 | <0.001 |
| Protein (g) | 11.4±0.8 | 25.6±1.4 | 8.3±0.8 | 9.5±0.7 | 15.9±1.6 | 8.6±1.1 | 0.072 | <0.001 | 0.811 | <0.001 |
| Fat (g) | 23.0±1.7 | 58.4±1.9 | 25.2±2.0 | 20.0±1.4 | 36.4±2.4 | 19.3±2.2 | 0.169 | <0.001 | 0.046 | <0.001 |
| Saturated fatty acids (g) | 9.6±0.7 | 21.1±0.9 | 10.0±0.8 | 8.2±0.6 | 7.7±0.8 | 7.7±0.9 | 0.186 | <0.001 | 0.046 | <0.001 |
| Carbohydrate (g) | 63.5±4.2 | 102.7±5.4 | 44.8±4.7 | 53.4±4.0 | 61.2±5.7 | 42.0±4.8 | 0.084 | <0.001 | 0.683 | <0.001 |
| **Dietary Intake ^‡^** |  |  |  |  |  |  |  |  |  |  |
| Energy (kcal) | 1583.4±47.0 | 1784.0±41.0 | 1695.5±41.5 | 1448.5±43.7 | 1546.8±58.1 | 1488.8±65.4 | 0.051 | 0.001 | 0.005 | 0.044 |
| Protein (g) | 48.8±1.7 | 61.0±2.0 | 57.2±1.6 | 51.8±2.0 | 55.0±2.0 | 52.7±2.3 | 0.262 | 0.029 | 0.110 | 0.072 |
| Fat (g) | 71.6±2.6 | 86.5±2.6 | 82.7±2.5 | 69.6±2.5 | 76.4±3.3 | 69.5±2.9 | 0.597 | 0.016 | 0.001 | 0.111 |
| Saturated fatty acids (g) | 22.0±1.0 | 31.7±1.0 | 30.2±1.0 | 27.0±1.2 | 29.2±1.2 | 26.1±1.3 | 0.002 | 0.106 | 0.015 | <0.001 |
| Carbohydrate (g) | 181,5±6,3 | 186,8±5,3 | 177,3±5,3 | 150,5±5,6 | 156,3±6,8 | 159,6±9.9 | <0.001 | <0.001 | 0.086 | 0.527 |

^†^Energy and macro nutrients intake from UPF, ^‡^Daily dietary intake. Data are presented the mean ± standard deviation. p^a^, p^b^, p^c^ : Independent sample t test were used. p^d^ :Repeated measure ANOVA. The model adjusted for age and BMI (kg/m^2^).
